# Associations between genetically predicted plasma protein levels and Alzheimer’s disease risk: a study using genetic prediction models

**DOI:** 10.1186/s13195-023-01378-4

**Published:** 2024-01-11

**Authors:** Jingjing Zhu, Shuai Liu, Keenan A. Walker, Hua Zhong, Dalia H. Ghoneim, Zichen Zhang, Praveen Surendran, Sarah Fahle, Adam Butterworth, Md Ashad Alam, Hong-Wen Deng, Chong Wu, Lang Wu

**Affiliations:** 1 Cancer Epidemiology Division, Population Sciences in the Pacific Program, University of Hawaii Cancer Center, University of Hawaii at Manoa, Honolulu, HI 96813 USA; 2 Laboratory of Behavioral Neuroscience, National Institute On Aging, Intramural Research Program, Baltimore, MD USA; 3 Department of Biostatistics, The University of Texas MD Anderson Cancer Center, Houston, TX USA; 4 MRC/BHF Cardiovascular Epidemiology Unit, Department of Public Health and Primary Care, University of Cambridge, Cambridge, UK; 5 NIHR Blood and Transplant Research Unit in Donor Health and Genomics, Department of Public Health and Primary Care, University of Cambridge, Cambridge, UK; 6 Tulane Center for Biomedical Informatics and Genomics., Division of Biomedical Informatics and Genomics, Deming Department of Medicine, Tulane University, 1440 Canal Street, New Orleans, LA 70112 USA; 7 Center for Outcomes Research, Ochsner Clinic Foundation, New Orleans, LA 70121 USA

**Keywords:** Genetic instrument, Protein biomarker, Alzheimer’s disease, Risk

## Abstract

**Background:**

Specific peripheral proteins have been implicated to play an important role in the development of Alzheimer’s disease (AD). However, the roles of additional novel protein biomarkers in AD etiology remains elusive. The availability of large-scale AD GWAS and plasma proteomic data provide the resources needed for the identification of causally relevant circulating proteins that may serve as risk factors for AD and potential therapeutic targets.

**Methods:**

We established and validated genetic prediction models for protein levels in plasma as instruments to investigate the associations between genetically predicted protein levels and AD risk. We studied 71,880 (proxy) cases and 383,378 (proxy) controls of European descent.

**Results:**

We identified 69 proteins with genetically predicted concentrations showing associations with AD risk. The drugs almitrine and ciclopirox targeting ATP1A1 were suggested to have a potential for being repositioned for AD treatment.

**Conclusions:**

Our study provides additional insights into the underlying mechanisms of AD and potential therapeutic strategies.

**Supplementary Information:**

The online version contains supplementary material available at 10.1186/s13195-023-01378-4.

## Summary box

### What is already know on this topic

There is one study evaluating associations between genetically predicted protein levels in dorsolateral prefrontal cortex and risk of Alzheimer’s disease (AD); another study focuses on 38 dementia-associated proteins to determine associations of their genetically predicted levels in plasma with AD risk; a third study assesses 184 cerebrospinal fluid proteins, 100 plasma proteins, and 27 brain proteins using protein quantitative trait loci as instruments for their associations with AD risk. Existing studies did not systematically evaluate associations of predicted levels proteins across the proteome in plasma using genetic prediction models, findings of which may identify novel proteins to confer translational perspective for risk assessment and therapeutic strategies of AD.

### What this study adds

Our study identifies 69 potential AD-associated proteins in plasma using comprehensive genetic prediction models as instruments. We also prioritize drugs almitrine and ciclopirox targeting ATP1A1 to have a potential for being repositioned for AD treatment.

### How this study might affect research, practice, or policy

The promising proteins identified in our study could be further investigated for their roles in AD risk assessment and therapeutic strategies.

## Introduction

Alzheimer’s disease (AD), the most common cause of dementia, has become a growing public health concern due to an unprecedented increase in life expectancy globally. In the USA, reported deaths from AD have increased 146.2% between 2000 and 2018, making it the sixth leading cause of death [1]. It is predicted that the annual cost of caring for AD patients will reach to a trillion dollars by 2050. AD is an irreversible and progressive disorder with neuropathological changes often occurring long before any symptom becomes apparent. The abnormal accumulation of amyloid-beta (Aβ) plaques, a hallmark of AD, is known to occur as early as two decades before the onset of clinical symptoms [2]. Abnormal phosphorylation of tau, the second canonical AD protein aggregate, is believed to occur shortly thereafter (15–20 years before symptom onset) [3]. While a great deal of research effort has focused on targeting pathological Aβ aggregates and tau neurofibrillary tangles, several drugs were approved by U.S. Food and Drug Administration (FDA), including Aduhelm® [4] and Leqembi® [5]. These approved drugs could relieve symptoms while whether they can cure AD relies on further analyses. As a result, it is critical to identify novel biomarkers and biological pathways that may contribute to AD risk.

Physiological changes that take place outside the brain (e.g., immune, vascular, and metabolic changes) have been shown to directly influence the function of neural cells and relate strongly to risk of developing AD [6, 7]. The identification of circulating peripheral proteins that drive the associations between peripheral biological changes and increased risk for AD may enhance our understanding of AD pathogenesis and thereby inform future therapeutic strategies. In addition to Aβ and tau, a number of proteins have also been recognized to be related to AD [8]. Translational and epidemiological research indicates that biological processes which operate outside of the central nervous system can contribute considerably to one’s risk of developing AD [6, 9]. These peripheral biological processes can be reflected in plasma and serum protein composition, i.e., secreted proteins. Identifying proteins that are causally associated with AD-relevant outcomes will deepen our understanding regarding how peripheral molecular changes, biological pathways, and regulatory mechanisms influence AD risk.

AD is highly heritable. Twin and family studies support that genetic factors could play a role in at least 80% of AD cases [10]. A recent genome-wide association study (GWAS) has identified 29 independent disease-associated risk loci by studying 71,880 (proxy) cases and 383,378 (proxy) controls of European ancestry [11]. The present study aimed at identifying novel protein biomarkers for AD through evaluating the associations between genetically predicted protein concentrations and AD risk, a design of proteome-wide association study (PWAS). Similar to the design of Mendelian randomization (MR) and transcriptome-wide association study (TWAS) [12–15], such a design can potentially reduce common biases imbedded in conventional epidemiological studies, such as selection biases, residual confounding, or reverse causality. We established and validated comprehensive protein genetic prediction models to fully capture the genetically regulated components of protein levels by using both *cis*- and *trans*-acting elements, thus providing higher statistical power than only using *cis*-acting elements alone (a common practice for related studies). We then related genetically predicted plasma concentrations to AD risk and, in doing so, causally implicated 69 circulating proteins in the AD pathogenesis, shedding light on the peripheral biology of AD.

## Methods

The genome and plasma proteome data of European descendants included in the INTERVAL study (subcohort 1 and subcohort 2) was used to establish and validate protein genetic prediction models. Detailed information about the INTERVAL study dataset has been described elsewhere [16]. In brief, participants were aged 18–80 and were generally in good health. The SOMAscan assay was used to measure the relative concentrations of 3620 plasma proteins or protein complexes. Quality control (QC) was performed at the sample and SOMAmer level. After excluding eight non-human protein targets, a total of 3283 SOMAmers remained for further study. DNA was used to assay ~ 830,000 variants on the Affymetrix Axiom UK Biobank genotyping array. Standard sample and variant QC was conducted, as described in the original publication [16]. SNPs were further phased using SHAPEIT3 and imputed using a combined 1000 Genomes Phase 3-UK10K reference panel via the Sanger Imputation Server, resulting in over 87 million imputed variants. Such SNPs were filtered using criteria of (1) imputation quality of at least 0.7, (2) minor allele frequency (MAF) of at least 5%, (3) Hardy–Weinberg equilibrium (HWE) *p* ≥ 5 × 10^−6^, (4) missing rates < 5%, and (5) presenting in the 1000 Genome Project data for European populations. In total, there were 4,662,360 variants passing these criteria.

In subcohort 1 (*N* = 2481), protein levels were log transformed and adjusted for age, sex, duration between blood draw and processing, and the first three principal components of ancestry. For the rank-inverse normalized residuals of each protein of interest, we followed the TWAS/FUSION framework [17] to develop genetic prediction models, using nearby SNPs (within 100 kb) of potentially associated SNPs as potential predictors. A false discovery rate (FDR) < 0.05 and *P*-value ≤ 5 × 10^−8^ were used to determine potentially associated SNPs in *cis-* and *trans-* regions, respectively. We defined *cis-*region as a region within 1 Mb of the transcriptional start site (TSS) of the gene encoding the target protein of interest. Subsequently, we extracted all SNPs located within 100 kb of the aforementioned potentially associated SNPs to serve as potential predictors for establishing protein prediction models, excluding any ambiguous SNPs. In order to include potential predictors from both *cis* and *trans* regions, we converted all the chromosome numbers to Z and combined them as a single pseudo chromosome. Four methods, namely, best linear unbiased predictor, elastic net, LASSO, and top1, were used for establishing the models. For developed protein prediction models with prediction performance (*R*^2^) of at least 0.01 [15, 18–23], which is a common threshold used in relevant studies, we further conducted external validation using subcohort 2 (*N* = 820) data. In brief, we generated predicted expression levels by applying the established protein prediction models to the genetic data, and then compared the predicted v.s. measured levels of each protein of interest. We selected proteins with a model prediction *R*^2^ of ≥ 0.01 in subcohort 1 and a correlation coefficient of ≥ 0.1 in subcohort 2 for the downstream association analysis.

To assess the associations between genetically predicted circulating protein levels and AD risk, we applied the validated protein prediction models to the summary statistics from a large GWAS meta-analysis of AD risk [24]. Instead of using the conventional approach of including clinically diagnosed AD alone, this GWAS combined clinically confirmed and parental diagnoses based by-proxy phenotypes, which has been demonstrated to confer great value in substantially increasing statistical power [25]. In brief, this study included a total of 85,934 cases (39,106 clinically diagnosed AD and 46,828 proxy AD) and 401,577 controls of European ancestry, which were obtained from various sources including The European Alzheimer & Dementia Biobank dataset (EADB), GR@ACE/DEGESCO study, The Rotterdam Study (RS1 and RS2), European Alzheimer’s Disease Initiative (EADI) Consortium, Genetic and Environmental Risk in AD (GERAD) Consortium/Defining Genetic, Polygenic, and Environmental Risk for Alzheimer’s Disease (PERADES) Consortium, The Norwegian DemGene Network, The Neocodex–Murcia study (NxC), The Copenhagen City Heart Study (CCHS), Bonn studies, and UK Biobank. Detailed information on study participants as well as genotyping and imputation methods for the samples from each of the included study can be found in the supplementary files of the original GWAS paper [24]. Risk estimates for the single marker association analyses were adjusted for sex, batch (if applicable), age (if applicable), and top principal components (PCs).

The TWAS/FUSION framework was used to determine the protein-AD associations, by leveraging correlation information between SNPs included in the prediction models from the phase 3, 1000 Genomes Project data of European ancestry [17]. We calculated the PWAS test statistic *Z*-score = *w'Z*/(*w'*Σ_s,s_*w*)^1/2^, where the *Z* is a vector of standardized effect sizes of SNPs for a given protein (Wald *z*-scores), *w* is a vector of prediction weights for the abundance feature of the protein being tested, and the *Σ*_s,s_ is the LD matrix of the SNPs estimated from the 1000 Genomes Project as the LD reference panel. The Bonferroni correction *P*-value < 0.05 was used to determine significant associations between genetically predicted protein concentrations and AD risk.

Ingenuity Pathway Analysis (IPA, Ingenuity System Inc, USA)) and Protein–Protein Interaction analysis via STRING database (version 12.0) with 0.400 confidence level [26] was implemented to cluster and classify enriched pathways for the identified proteins using default interaction resources, including Textmining, Experiments, Databases, Co-expression, Neighborhood, Gene Fusion, and Co-occurrence. We also investigated potentially repositionable drugs targeting the genes encoding associated proteins, by using the GREP (Genome for REPositioning drugs) tool [27]. We further conducted molecular docking analysis considering ATP1A1 protein as the drug target protein and almitrine and ciclopirox as the drug agents [28].

## Results

**Fig. 1:**
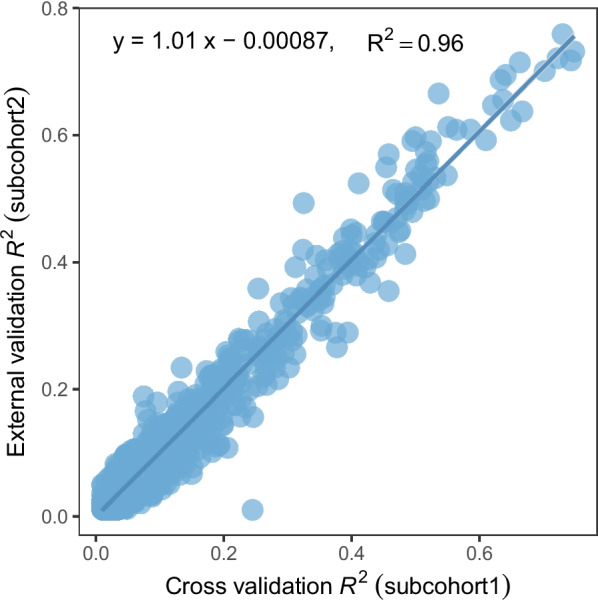
Performance of protein expression prediction models in INTERVAL subcohort1 and subcohort2 datasets for proteins showing internal and external validation performance of *R*^2^ ≥ 0.01

**Fig. 2:**
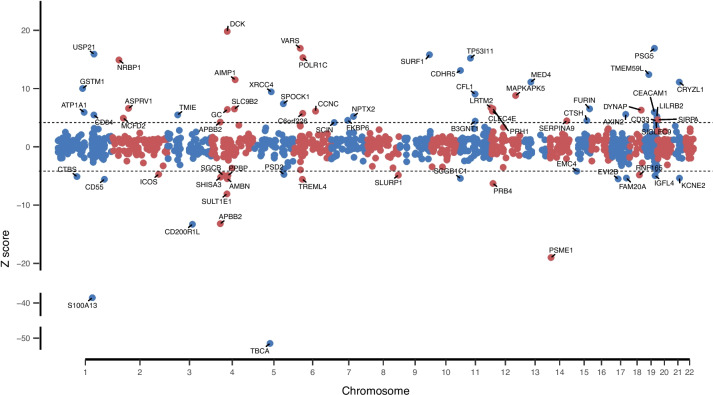
Associations Z scores for proteins showing an association at Bonferroni corrected *P*-value ≤ 0.05 with AD risk

In this study, potential predictors were identified for 1870 proteins, and protein prediction models were successfully established for 1864 proteins. For the 1413 of the remaining proteins, there was no SNP showing an association at FDR < 0.05 for *cis* SNPs and *P*-value ≤ 5 × 10^−8^ for *trans* SNPs. After internal and external validation, there were 1389 proteins showing internal and external validation performance of *R*^2^ ≥ 0.01. The median external validation *R*^2^ was 0.06. There were 459, 189, and 38 proteins that showed external validation *R*^2^ ≥ 0.1, 0.2, and 0.5, respectively. Overall, proteins that could be predicted well in INTERVAL subcohort 1 also tended to be predicted well in subcohort 2 in external validation analyses (a correlation coefficient of 0.96 for *R*^2^ in two data sets; Fig. 1). Using the TWAS/FUSION framework, we examined the association for a total of 1340 proteins. For the remaining 49 proteins, more than half of the SNPs included in the models were not present in the AD GWAS summary; therefore, their associations with AD risk were not considered. We identified 69 proteins with genetically predicted concentrations showing associations with AD risk after Bonferroni correction (*P*-value < 3.01 × 10^−5^) (Table 1; Fig. 2). Of those 69 proteins, positive associations were observed for 45 of them, and inverse associations were observed for 24 (Table 1; Fig. 2).

**Table 1:** Proteins showing a significant association with Alzheimer’s disease risk for their genetically predicted concentrations in plasma

Protein	Protein full name	SOMAMER ID	Encoding gene ID	Modeling method^a^	Num of predicting SNPs in model	Num of predicting SNPs-Cis^b^	Num of predicting SNPs-Trans	Model internal cross validation *R*^2^	Model external validation *R*^2^	Z score^c^	*P*-value	Distance of gene to closest risk SNP (kb)^d^
TBCA	Tubulin-specific chaperone A	TBCA.12501.10.3	TBCA	blup	470	0	470	0.54	0.51	− 50.90	< 1.09 × 10^−298^	9,236.20
S100A13	Protein S100-A13	S100A13.7223.60.3	S100A13	blup	528	0	528	0.56	0.54	− 39.10	< 1.09 × 10^−298^	7,564.12
DCK	Deoxycytidine kinase	DCK.9836.20.3	DCK	lasso	5	0	5	0.04	0.02	19.80	7.23 × 10^−87^	31,660.52
PSME1	Proteasome activator complex subunit 1	PSME1.5918.5.3	PSME1	lasso	13	0	13	0.19	0.11	− 19.00	8.12 × 10^−81^	28,693.45
SYVC	Valine–tRNA ligase	VARS.13083.18.3	VARS	enet	69	0	69	0.38	0.27	16.90	2.71 × 10^−64^	833.24
PSG5	Pregnancy-specific beta-1-glycoprotein 5	PSG5.9314.9.3	PSG5	enet	50	16	34	0.07	0.05	16.90	8.68 × 10^−64^	1,740.05
UBP21	Ubiquitin carboxyl-terminal hydrolase 21	USP21.12681.63.3	USP21	enet	39	0	39	0.14	0.08	15.90	3.69 × 10^−57^	26.10
SURF1	Surfeit locus protein 1	SURF1.8009.121.3	SURF1	enet	68	0	68	0.06	0.05	15.80	1.62 × 10^−56^	28,552.64
RPAC1	DNA-directed RNA polymerases I and III subunit RPAC1	POLR1C.12939.1.3	POLR1C	enet	44	0	44	0.03	0.01	15.30	6.67 × 10^−53^	2,542.63
P5I11	Tumor protein p53-inducible protein 11	TP53I11.13022.20.3	TP53I11	enet	19	0	19	0.06	0.05	15.20	2.25 × 10^−52^	2,426.44
NRBP	Nuclear receptor-binding protein	NRBP1.12616.45.3	NRBP1	enet	40	11	29	0.07	0.02	14.90	3.56 × 10^−50^	9,881.28
MO2R2	Cell surface glycoprotein CD200 receptor 2	CD200R1L.8980.19.3	CD200R1L	top1	1	0	1	0.09	0.03	− 13.30	4.44 × 10^−40^	42,252.95
APBB2	Amyloid beta A4 precursor protein-binding family B member 2	APBB2.12753.6.3	APBB2	top1	1	0	1	0.05	0.03	− 13.20	8.33 × 10^−40^	613.20
MUCDL	Cadherin-related family member 5	CDHR5.9962.1.3	CDHR5	top1	1	0	1	0.03	0.02	13.10	2.66 × 10^−39^	46,763.76
TM59L	Transmembrane protein 59-like	TMEM59L.9959.60.3	TMEM59L	enet	47	0	47	0.05	0.01	12.40	2.48 × 10^−35^	16,869.43
EMAP-2	Endothelial monocyte-activating polypeptide 2	AIMP1.2714.78.2	AIMP1	lasso	4	0	4	0.06	0.03	11.50	1.51 × 10^−30^	67,037.86
MED4	Mediator of RNA polymerase II transcription subunit 4	MED4.14021.81.3	MED4	top1	1	0	1	0.09	0.05	11.10	7.48 × 10^−29^	NA^**d**^
QORL1	Quinone oxidoreductase-like protein 1	CRYZL1.9207.60.3	CRYZL1	enet	18	0	18	0.16	0.13	11.10	7.76 × 10^−29^	7,440.72
GST M1-1	Glutathione S-transferase Mu 1	GSTM1.7239.9.3	GSTM1	enet	20	6	14	0.03	0.05	10	1.03 × 10^−23^	342.01
XRCC4	DNA repair protein XRCC4	XRCC4.9886.28.3	XRCC4	lasso	4	0	4	0.05	0.03	9.43	3.98 × 10^−21^	3,849.83
Cofilin-1	Cofilin-1	CFL1.4203.50.2	CFL1	lasso	6	0	6	0.11	0.08	9.04	1.59 × 10^−19^	5,600.20
MAPK5	MAP kinase-activated protein kinase 5	MAPKAPK5.8382.47.3	MAPKAPK5	lasso	21	0	21	0.48	0.49	8.79	1.48 × 10^−18^	1,439.76
SULT 1E	Estrogen sulfotransferase	SULT1E1.9878.3.3	SULT1E1	lasso	10	0	10	0.11	0.05	− 8.08	6.54 × 10^−16^	30,508.08
Testican-1	Testican-1	SPOCK1.5490.53.3	SPOCK1	enet	24	0	24	0.05	0.01	7.38	1.63 × 10^−13^	14,121.40
LRTM2	Leucine-rich repeat and transmembrane domain-containing protein 2	LRTM2.8906.60.3	LRTM2	enet	49	0	49	0.04	0.01	6.70	2.03 × 10^−11^	111,790.06
APRV1	Retroviral-like aspartic protease 1	ASPRV1.13023.8.3	ASPRV1	enet	42	0	42	0.14	0.12	6.59	4.26 × 10^−11^	32,627.91
PRPC	Salivary acidic proline-rich phosphoprotein 1/2	PRH1.10502.15.3	PRH1	enet	38	21	17	0.05	0.04	6.53	6.52 × 10^−11^	102,686.22
Furin	Furin	FURIN.6276.16.3	FURIN	enet	43	0	43	0.07	0.05	6.51	7.76 × 10^−11^	12,182.62
SL9B2	Mitochondrial sodium/hydrogen exchanger 9B2	SLC9B2.9088.20.3	SLC9B2	lasso	4	0	4	0.02	0.02	6.48	9.02 × 10^−11^	63,740.34
Gc-Globulin, Mixed Type	Vitamin D-binding protein	GC.6581.50.3	GC	enet	30	0	30	0.17	0.16	6.40	1.53 × 10^−10^	32,408.56
PRB4	Basic salivary proline-rich protein 4	PRB4.12590.67.3	PRB4	top1	1	0	1	0.02	0.03	− 6.29	3.21 × 10^−10^	102,259.78
CR026	Dynactin-associated protein	DYNAP.10692.48.3	DYNAP	top1	1	0	1	0.02	0.02	6.29	3.21 × 10^−10^	3,934.47
CCNC	Cyclin-C	CCNC.7817.36.3	CCNC	top1	1	0	1	0.02	0.03	6.11	1.01 × 10^−9^	14,622.63
CLC4E	C-type lectin domain family 4 member E	CLEC4E.7077.9.4	CLEC4E	top1	1	0	1	0.02	0.02	6.11	1.01 × 10^−9^	105,033.89
CEACAM1	Carcinoembryonic antigen-related cell adhesion molecule 1	CEACAM1.8031.11.3	CEACAM1	enet	21	0	21	0.23	0.26	5.94	2.91 × 10^−9^	2,400.48
AT1A1	Sodium/potassium-transporting ATPase subunit alpha-1	ATP1A1.11993.227.3	ATP1A1	enet	15	0	15	0.06	0.07	5.91	3.52 × 10^−9^	7,027.43
CF226	Uncharacterized protein C6orf226	C6orf226.8078.15.3	C6orf226	lasso	7	0	7	0.07	0.07	5.74	9.61 × 10^−9^	1,915.81
AXIN2	Axin-2	AXIN2.8429.16.3	AXIN2	enet	18	0	18	0.40	0.44	5.57	2.48 × 10^−8^	1,978.90
TRML4	Trem-like transcript 4 protein	TREML4.11139.4.3	TREML4	lasso	9	0	9	0.21	0.20	− 5.57	2.51 × 10^−8^	253.89
DAF	Complement decay-accelerating factor	CD55.5069.9.3	CD55	lasso	11	11	0	0.11	0.07	− 5.57	2.55 × 10^−8^	197.03
EVI2B	Protein EVI2B	EVI2B.13028.2.3	EVI2B	enet	11	0	11	0.07	0.05	− 5.52	3.30 × 10^−8^	11,571.33
TMIE	Transmembrane inner ear expressed protein	TMIE.7992.3.3	TMIE	enet	7	0	7	0.04	0.05	5.47	4.46 × 10^−8^	10,490.88
AMBN	Ameloblastin	AMBN.6522.57.3	AMBN	lasso	11	0	11	0.15	0.07	− 5.47	4.52 × 10^−8^	31,259.13
SLAF5	SLAM family member 5	CD84.8770.136.3	CD84	blup	46	0	46	0.05	0.08	5.46	4.65 × 10^−8^	644.50
SG1C1	Secretoglobin family 1C member 1	SCGB1C1.5960.49.3	SCGB1C1	enet	25	0	25	0.18	0.10	− 5.42	5.84 × 10^−8^	47,187.26
KCNE2	Potassium voltage-gated channel subfamily E member 2	KCNE2.10427.2.3	KCNE2	enet	33	0	33	0.26	0.26	− 5.38	7.55 × 10^−8^	8,215.37
FA20A	Pseudokinase FAM20A	FAM20A.6433.57.3	FAM20A	enet	30	17	13	0.04	0.03	− 5.36	8.43 × 10^−8^	4,985.48
SHSA3	Protein shisa-3 homolog	SHISA3.7057.18.3	SHISA3	enet	22	0	22	0.23	0.16	− 5.20	2.01 × 10^−7^	2,200.66
NPTX2	Neuronal pentraxin-2	NPTX2.6521.35.3	NPTX2	enet	30	0	30	0.04	0.05	5.20	2.01 × 10^−7^	1,685.45
ILT-4	Leukocyte immunoglobulin-like receptor subfamily B member 2	LILRB2.5633.65.3	LILRB2	lasso	8	6	2	0.21	0.22	5.19	2.15 × 10^−7^	6.22
DIAC	Di-N-acetylchitobiase	CTBS.6115.40.3	CTBS	enet	20	0	20	0.14	0.09	− 5.11	3.23 × 10^−7^	24,873.14
IGFL4	Insulin growth factor-like family member 4	IGFL4.6353.60.3	IGFL4	enet	18	0	18	0.10	0.05	− 4.95	7.47 × 10^−7^	300.60
NAP-2	Neutrophil-activating peptide 2	PPBP.2790.54.2	PPBP	enet	22	0	22	0.03	0.01	− 4.93	8.04 × 10^−7^	34,653.31
MCFD2	Multiple coagulation factor deficiency protein 2	MCFD2.10476.23.3	MCFD2	blup	744	0	744	0.53	0.60	4.92	8.62 × 10^−7^	9,597.07
SLUR1	Secreted Ly-6/uPAR-related protein 1	SLURP1.6401.73.3	SLURP1	lasso	4	0	4	0.06	0.03	− 4.86	1.17 × 10^−6^	1,285.78
SGCB	Beta-sarcoglycan	SGCB.7034.4.3	SGCB	enet	16	0	16	0.10	0.06	− 4.84	1.30 × 10^−6^	12,688.03
RN165	RING finger protein 165	RNF165.11561.32.3	RNF165	enet	18	0	18	0.15	0.11	− 4.84	1.32 × 10^−6^	12,275.48
PSD2	PH and SEC7 domain-containing protein 2	PSD2.9118.7.3	PSD2	enet	43	0	43	0.15	0.16	− 4.74	2.14 × 10^−6^	11,257.00
ICOS	Inducible T-cell costimulator	ICOS.14084.191.3	ICOS	enet	27	0	27	0.17	0.12	− 4.72	2.41 × 10^−6^	1,058.05
SHPS1	Tyrosine-protein phosphatase non-receptor type substrate 1	SIRPA.5430.66.3	SIRPA	blup	2,563	2,506	57	0.63	0.62	4.63	3.71 × 10^−06^	1,480.84
FKBP6	Inactive peptidyl-prolyl cis–trans isomerase FKBP6	FKBP6.12529.32.3	FKBP6	blup	1,593	0	1,593	0.49	0.53	4.54	5.53 × 10^−6^	17,800.84
Cathepsin H	Cathepsin H	CTSH.8465.52.3	CTSH	lasso	13	12	1	0.30	0.28	4.51	6.58 × 10^−6^	15.80
Siglec-3	Myeloid cell surface antigen CD33	CD33.3166.92.1	CD33	enet	98	92	6	0.41	0.45	4.47	7.78 × 10^−6^	0.37
SPA9	Serpin A9	SERPINA9.7266.4.3	SERPINA9	lasso	4	0	4	0.02	0.02	4.46	8.02 × 10^−6^	1,990.20
B3GN1	Beta-1,4-glucuronyltransferase 1	B3GNT1.8259.25.3	B3GNT1	enet	50	0	50	0.15	0.17	4.40	1.09 × 10^−5^	6,090.90
APBB2	Amyloid beta A4 precursor protein-binding family B member 2	APBB2.12761.12.3	APBB2	lasso	9	6	3	0.03	0.01	4.24	2.26 × 10^−5^	613.20
TMM85	ER membrane protein complex subunit 4	EMC4.13516.46.3	EMC4	enet	73	0	73	0.21	0.20	− 4.20	2.63 × 10^−5^	24,505.40
Siglec-9	Sialic acid-binding Ig-like lectin 9	SIGLEC9.3007.7.2	SIGLEC9	blup	618	580	38	0.72	0.73	4.19	2.75 × 10^−5^	99.83
ADSV	Adseverin	SCIN.12684.5.3	SCIN	lasso	4	0	4	0.03	0.02	4.17	3.01 × 10^−5^	340.75

^a^enet: elastic net; blup: best linear unbiased predictor

^b^SNPs within 1 MB of the protein-encoding gene

^c^*Z* score represents the direction of the association between genetically predicted protein levels and AD risk

^d^Risk SNPs identified in previous GWAS or fine-mapping studies. The SNP list is included in Table S1

^e^NA indicates no risk SNP was reported on the chromosome

For those proteins associated with AD risk, the Core Analysis was performed in Ingenuity Pathway Analysis. Assembly of RNA Polymerase I Complex and DNA Double-Strand Break Repair by Non-Homologous End Joining were two canonical pathways showing significant enrichments at *P* < 0.05 (Table S2; Figure S1). In the Network Analysis, Cell-To-Cell Signaling and Interaction, Hematological System Development and Function, Immune Cell Trafficking was identified which involved 19 associated proteins (Table S3; Figure S2). Based on the Disease and Biological Functions analysis, the top disease functional categories identified were shown in Table S4.

Protein interactions of 69 associated proteins were investigated using the STRING database (Figure S3). In the network, five proteins (ILT-4, PRPC, SHPS1, Siglec-3, and Siglec-9) had three or more interactions with other proteins. Among them, Siglec-3 (known as CD33) was reported as a risk factor for AD and both the mRNA level and protein abundance were found to be increased in AD patients compared to the age-matched controls [29]. This finding is consistent with our current study (*Z*-score = 4.47, *P*-value = 7.78 × 10^−6^).

Based on The Anatomical Therapeutic Chemical (ATC) test using GREP, the drugs almitrine and ciclopirox targeting ATP1A1 were suggested to have a potential for being repositioned for AD treatment (odds ratio (OR) = 63.0; *P* = 0.022 for almitrine; OR = 35.9, *P* = 0.035 for ciclopirox).

**Fig. 3:**
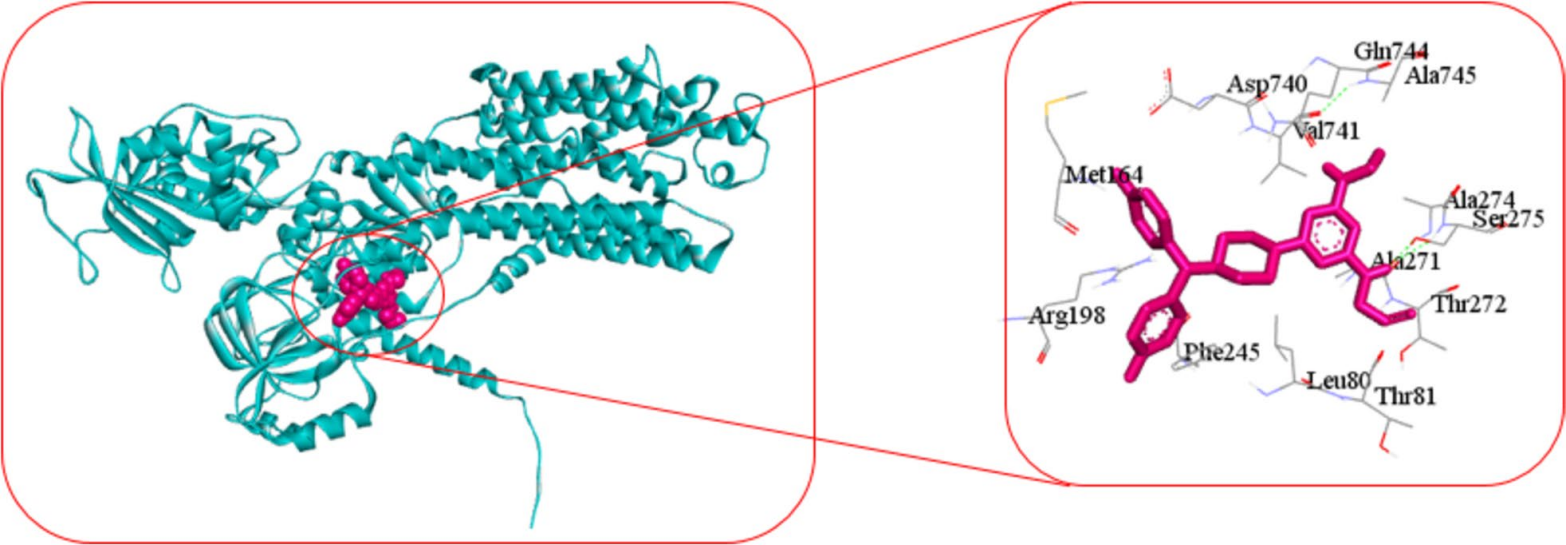
The 3D structure (left) and 2D schematic diagram (right) of the ATP1A1 potential target and almitrine drug

**Fig. 4:**
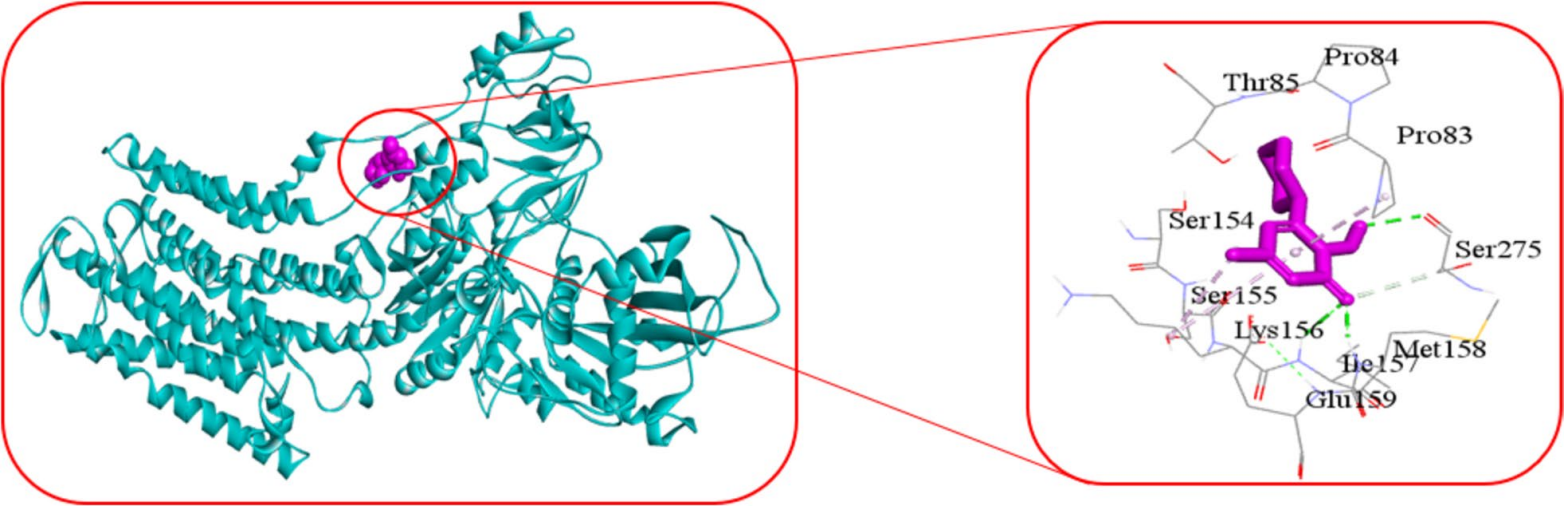
The 3D structure (left) and 2D schematic diagram (right) of the ATP1A1 potential target and ciclopirox

For molecular docking analysis, we downloaded the 3D structure of ATP1A1 protein from Protein Data Bank (PDB) with source code 3KDP and almitrine and ciclopirox drug from the PubChem database [30, 31]. AutoDock-Vina produced − 7.6 kcal/mol binding energy for ATP1A1 protein with almitrine drug agent and − 6.2 kcal/mol binding energy for ATP1A1 protein with ciclopirox drug agent. Figure 3 showed the 3D structure (left) and 2D schematic diagram (right) of the ATP1A1 potential target and almitrine drug with interacting amino acids: Leu80, Thr81, Met164, Arg198, Phe245, Ala271, Thr272, Ala274, Ser275, Asp740, Val741, Gln744, and Ala745. Figure 4 showed the 3D structure (left) and 2D schematic diagram (right) of the ATP1A1 potential target and ciclopirox drug.

## Discussion

To our knowledge, the present study is the first large population-based study to systematically investigate the associations between genetically predicted circulating protein concentrations in plasma and AD risk using genetic instruments of comprehensive protein prediction models. Overall, we identified 69 proteins that were significantly associated with AD risk after Bonferroni correction. If validated in future studies, our findings could add substantial new knowledge to the etiology of AD and provide a list of protein markers to facilitate precision preventive or therapeutic trials of AD.

Recently, plasma proteins including Aß_42_ and phosphorylated tau (p-tau217, p-tau181, and others) have been identified as promising plasma biomarkers for clinically and pathologically defined AD [32–34]. While these biomarkers will be incredibly useful for participant risk stratification, it remains vitally important to identify additional AD biomarkers to further understand the pathophysiological processes leading to AD. By examining associations of genetically predicted protein levels in plasma with AD risk, we are able to go beyond a traditional examination of protein-AD association and begin to understand whether proteins may be causally relevant. For example, although plasma levels of YKL-40 [35] have been associated with AD, we did not observe evidence of an association for genetically predicted levels of YKL-40 (*Z* = 1.50; *P* = 0.13). This finding seems to support that although specific proteins such as YKL-40 could be strong biomarkers, they may not be causally relevant.

We identified multiple AD-associated proteins using proteomic and genetic methods that were reported for the first time (Table 1). For some of them, there is already existing evidence from functional work supporting their potential links with AD. For example, cofilin-1, as a major actin depolymerizer in the central nervous system, plays a crucial role in maintaining the structure and proper function of neurons [36]. Cofilin rods, which are primarily composed of actin and cofilin-1 and form in response to stressing conditions, have been suggested to be associated with neurodegenerative diseases such as AD by disrupting dendritic transportation and inducing synaptic dysfunction [36, 37]. Additional research is warranted to understand the identified associations for the other proteins.

By using GREP, the drugs almitrine and ciclopirox were suggested to be potentially repositionable for AD treatment. A double-blind controlled study involving patients with memory loss, lack of concentration, impaired mental alertness, and emotional instability supported that almitrine-raubasine could improve cognitive impairments [35]. Another controlled multicenter study investigating patients with cognitive decline (assessed by MMSE, SCAG) again suggested almitrine-raubasine significantly improved symptomatology compared with placebo [38]. Three other trails conducted in China involving 206 patients with vascular dementia also supported significant beneficial effect of almitrine-raubasine combination on the improvement of cognitive function measured by MMSE [39], although high risk of bias was observed. Other research supported that ciclopirox could protect neuronal cells from cell death and astrocytes from peroxynitrate toxicity [40, 41]. Future work may be warranted to further investigate whether almitrine and ciclopirox can indeed treat AD.

The strengths of our study include a high statistical power to identify AD-associated proteins given the large sample size in the main association analysis. Instead of merely using individual protein quantitative trait loci (pQTL) as instruments, we developed comprehensive protein genetic prediction models using a state-of-the-art method and externally validated their performance before applying them to downstream association tests. Our previous work has supported that compared with individual QTLs, comprehensive prediction models can better capture genetically regulated components of molecular levels and thus further increase statistical power [42]. In two recently published studies, pQTLs in plasma were used to assess proteins potentially associated with AD risk [43, 44]. It is expected that the current work should have improved power as well as scope compared with these two existing studies. Particularly, in Walker et al. [44], only proteins showing an association for the directly measured levels were tested. In Yang et al. [43], a relatively smaller dataset (*n* = 636) was used to determine plasma pQTLs. Correspondingly, a smaller number of pQTLs for 127 proteins were identified for association analyses. In Wingo et al. [45], prediction models for 376 proteins in brain tissue were established, and 13 proteins were identified to be associated with AD risk. It is also worth noting that in the previous studies, AD GWAS summaries involving a less number of cases and controls were employed. Walker and Yang utilized the GWAS summary data from the Kunkle study [46], comprising 21,982 clinically diagnosed AD cases and 41,944 cognitively normal controls, while Wingo employed the AD GWAS summary data from the Jansen study [11], encompassing 71,880 cases (clinically diagnosed AD and AD-by-proxy) and 383,378 controls. In the present study, we utilized a more comprehensive GWAS summary data from a more recent study, including 85,934 cases (comprising 39,106 clinically diagnosed AD and 46,828 proxy AD) and 401,577 controls. We checked the associations of the proteins reported in these previous studies in the current work. Interestingly, only three of the reported proteins showed consistent associations (same effect direction and nominal *P*-value < 0.05) in the current work (Table S5). To further examine the robustness of these results, we extended our examination by using two independent protein genetic prediction models established by others using independent methods, namely Atherosclerosis Risk in Communities (ARIC) European ancestry models [47] and INTERVAL *cis-*models [48]. Notably, when we focused only on plasma, a majority of the examined proteins did not exhibit significant associations with the risk of AD when using either ARIC European ancestry or INTERVAL *cis*-models. This observation that aligns well with results based on our developed models suggests that these prior findings could potentially be false positives. Again, such a discrepancy could be potentially attributed to the relatively limited utility of individual pQTL SNPs in fully elucidating the genetically regulated components of protein levels. Further studies are warranted to better characterize the other previously reported proteins.

Several limitations of the current work also need to be acknowledged. First, our findings may be subject to potential pleiotropic effects, limiting the ability to draw causal insights. Second, given the nature of our study of using genetic instruments to predict plasma protein levels, we are only able to capture the genetically regulated components of the protein concentrations, without incorporating the components influenced by exogenous exposures. Like the concept of transcriptome-wide association studies (TWAS), our proteome-wide association study (PWAS) aims to investigate the relationship between the genetically determined components of protein levels and disease risk. Further prospective studies with measured protein levels in pre-disease plasma samples are needed to better evaluate the relationship. Finally, when we establish genetic models to estimate such genetically determined components of protein levels, we carefully controlled for age, sex, duration between blood draw and processing, and top genetic principal components. However, we acknowledge that specific factors such as smoking and body mass index (BMI) were not controlled for during model construction using the INTERVAL dataset due to a lack of relevant data available to us [49]. Future studies are in need to validate our findings.

In conclusion, in this large association study using genetic instruments, we identified multiple novel AD risk-associated proteins. If validated with further investigations, our study may add additional knowledge to the underlying mechanisms of AD.

### Supplementary Information

**Additional file 1: Figure S1.** Enriched canonical pathways for the identified associated proteins. The and p-value below each term indicates the significance level of each pathway. **Figure S2.** The network was identified by Ingenuity Pathway Analysis (IPA). A solid line represents a direct interaction between two nodes and a dotted line indicates an indirect interaction. **Figure S3.** Network nodes represent proteins and edges represent protein-protein associations.

**Additional file 2: Table S1.** Risk SNPs identified to be associated with AD risk in previous GWAS or fine-mapping studies. **Table S2.** Ingenuity Canonical Pathways. **Table S3.** Network analysis. **Table S4.** Disease and Biological Functions analysis. **Table S5.** Association results of the proteins reported in previous studies.

## Data Availability

Summary statistics of the GWAS meta-analysis of AD risk by Bellenguez et al. are available at GWAS Catalog (https://www.ebi.ac.uk/gwas/) under accession no. GCST90027158. For the INTERVAL SomaLogic study, the individual-level genotype and protein data, and full summary association results from the genetic analysis, are available through the European Genotype Archive (accession number EGAS00001002555). Summary association results are also publicly available at http://www.phpc.cam.ac.uk/ceu/proteins/, through PhenoScanner (http://www.phenoscanner.medschl.cam.ac.uk) and from the NHGRI-EBI GWAS Catalog (https://www.ebi.ac.uk/gwas/downloads/summary-statistics). The scripts and protein genetic prediction models are available at https://github.com/Arthur1021/Protein-prediction-models.
