# Supplementary material for: Associations between genetically predicted plasma protein levels and Alzheimer’s disease risk: a study using genetic prediction models
Source: Alzheimers Res Ther. 2024 Jan 11;16:8. doi: 10.1186/s13195-023-01378-4 (PMC10782590; doi:10.1186/s13195-023-01378-4)
Supplement: Supplementary file 1 — Additional file 1: Figure S1. Enriched canonical pathways for the identified associated proteins. The and p-value below each term indicates the significance level of each pathway. Figure S2. The network was identified by Ingenuity Pathway Analysis (IPA). A solid line represents a direct interaction between two nodes and a dotted line indicates an indirect interaction. Figure S3. Network nodes represent proteins and edges represent protein-protein associations. [file 13195_2023_1378_MOESM1_ESM.docx]

**Supplementary Materials**

**
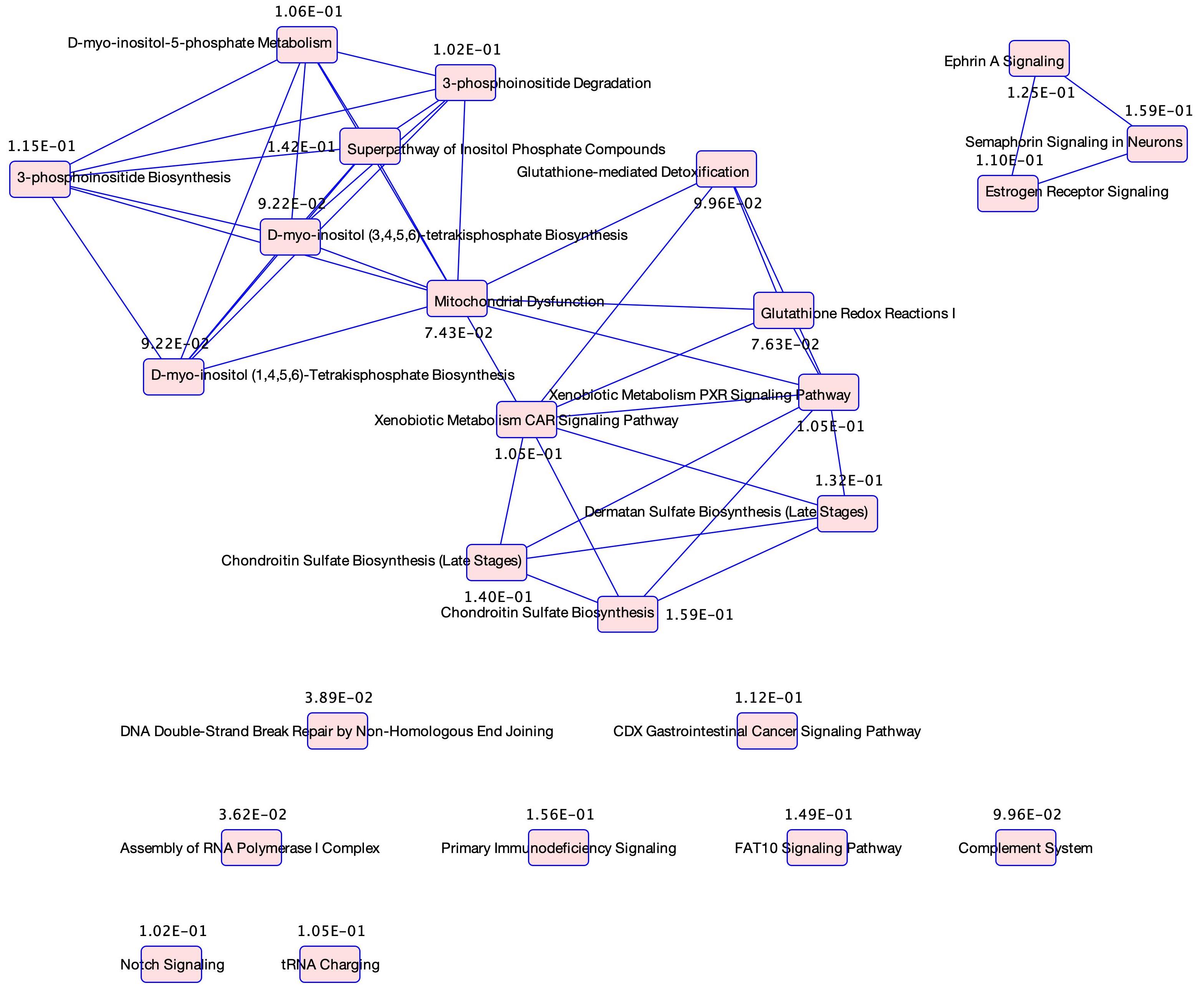
**

**Figure S1**. Enriched canonical pathways for the identified associated proteins. The and *p*-value below each term indicates the significance level of each pathway.


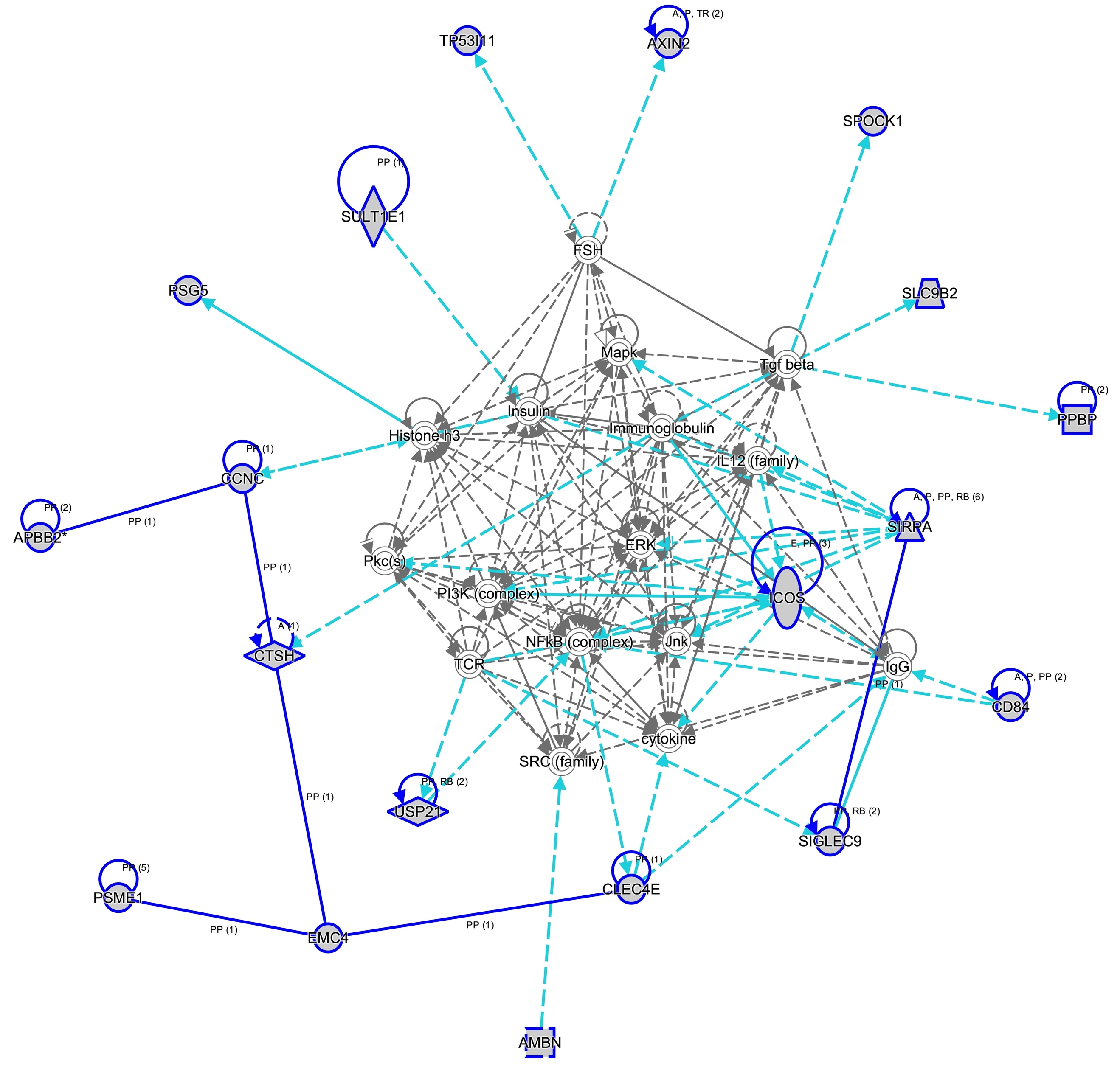


**Figure S2**. The network was identified by Ingenuity Pathway Analysis (IPA). A solid line represents a direct interaction between two nodes and a dotted line indicates an indirect interaction.


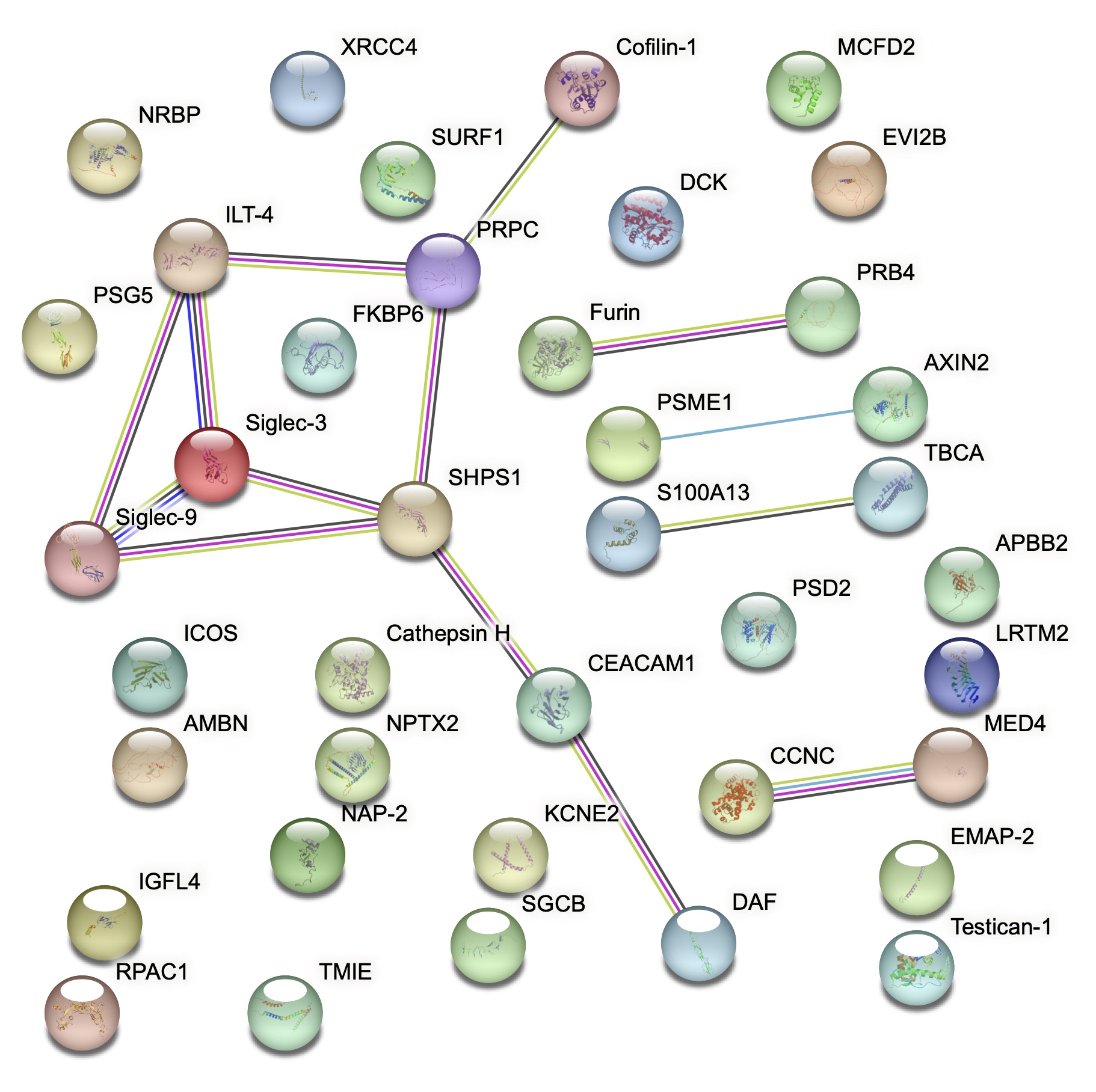


**Figure S3**. Network nodes represent proteins and edges represent protein-protein associations.
